# Editorial: Sarcopenia and nutrition in chronic kidney disease

**DOI:** 10.3389/fnut.2026.1788050

**Published:** 2026-01-29

**Authors:** Barbara Perez Vogt, Maryanne Zilli Canedo Silva, Heitor S. Ribeiro

**Affiliations:** 1 Medicine Faculty, Federal University of Uberlandia, Uberlândia, Brazil; 2 Internal Medicine Department, Botucatu Medical School, São Paulo State University, UNESP, Botucatu, Brazil; 3 Faculty of Health Sciences, University of Brasilia, Brasília, Brazil

**Keywords:** kidney replacement therapy, muscle strength, muscle wasting, physical activity, sarcopenia

Sarcopenia is a neuromuscular disease characterized by low muscle strength and muscle mass. Although initially described as a geriatric syndrome, it is now recognized as a clinically relevant condition in chronic diseases, including chronic kidney disease (CKD) (1). Patients with CKD are at increased risk of low levels of muscle mass and strength and, consequently, of developing sarcopenia (2).

In individuals undergoing dialysis, the pathophysiology of sarcopenia is complex and multifactorial, involving both disease and dialysis-related mechanisms. These include chronic inflammation, oxidative stress, hormonal imbalances, metabolic acidosis, uremia-induced protein-energy wasting, the physical and psychological burden of dialysis therapy, the presence of comorbidities, and reduced levels of physical activity (3). In addition, sarcopenia in CKD is influenced by demographic factors, comorbidity profiles, nutritional status and body composition, biochemical and metabolic markers, as well as treatment-related variables (4).

In the current Research Topic, three studies addressed potential risk factors associated with sarcopenia in patients undergoing hemodialysis (HD). Liu et al. observed that patients on HD in southwest China with iron deficiency exhibited significantly lower handgrip strength compared with those without deficiency. No differences were found in muscle mass, except in a subgroup analysis among patients with overweight (β = 0.50, 95% CI: 0.17; 0.84, *p* = 0.004). Consistently, a Brazilian cohort study showed that reduced muscle strength (dynapenia) was independently associated with fatigue in patients on HD, regardless of the dynapenia phenotype (handgrip strength and/or sit-to-stand test) (Duarte et al.). Moreover, another study showed that non-leisure-time physical activity was significantly associated with a reduced risk of developing sarcopenia (RR = 0.449, 95% CI: 0.248–0.814), reinforcing the protective role of an active lifestyle in patients on HD (Chang et al.).

In light of the burden of sarcopenia and its multifactorial determinants, appropriate screening and diagnosis are essential in patients with CKD and those undergoing dialysis. Despite its high prevalence (2, 5), the definition and methods for sarcopenia screening and diagnosis vary, mainly due to disease-specificities, and there is no consensus on methods for assessment in this population (2). For screening, SARC-F and SARC-CalF are the most used tools. However, in some settings, the development of predictive models for muscle health decline in patients with CKD has been proposed to identify those at high risk, facilitating prompt interventions. Lu et al. explored the data from the China Health and Retirement Longitudinal Study (CHARLS) cohort to develop and validate a sarcopenia risk prediction model for patients with CKD. The final model identified waist circumference, age, low-density lipoprotein (LDL-C), triglycerides (TG), high-density lipoprotein (HDL-C), and diastolic blood pressure as significant predictors of sarcopenia in this population.

Once individuals at risk are identified, the diagnosis of sarcopenia relies on standardized operational criteria. According to the revised consensus of the European Working Group on Sarcopenia in Older People (EWGSOP2), low muscle strength is a hallmark of sarcopenia, with low muscle quantity and quality used to confirm the sarcopenia diagnosis, and identifies poor physical performance as indicative of severe sarcopenia (1). For muscle mass assessments, different methods have been applied. Zheng et al. evaluated in a meta-analysis the use of the creatinine-to-cystatin C ratio and observed that this ratio could serve as a valuable tool for evaluating muscle mass, as well as an indicator of nutritional status, linking sarcopenia to poor prognosis in patients with CKD.

Other markers of muscle mass have been proposed, such as the adductor pollicis muscle thickness. However, in patients on HD, this anthropometric measurement was not associated with physical function assessed by five different methods, muscle mass estimated by bioelectrical impedance, and nutritional status evaluated by the Malnutrition Inflammation Score (Morais et al.).

Malnutrition is a contributing factor to the development of sarcopenia, as it promotes skeletal muscle loss through weight reduction, protein and micronutrient deficiencies, which are essential for muscle function and hormonal regulation. Chronic inflammation further exacerbates malnutrition by inducing anorexia, reducing dietary intake, and increasing resting energy expenditure, thereby accelerating muscle catabolism. Additionally, malnourished individuals frequently present impaired physical performance and reduced physical activity, which contribute to muscle atrophy and potentiate the progression of sarcopenia (6).

The coexistence of malnutrition and sarcopenia, referred to as malnutrition-sarcopenia syndrome, has been associated with adverse outcomes, as described by Wang et al. The authors reported that malnutrition-sarcopenia syndrome is prevalent among patients on HD and represents a combined risk factor for vascular calcification and major adverse cardiovascular events, which include cardiovascular mortality or hospitalization due to acute myocardial infarction, acute heart failure, or stroke.

Similarly, protein-energy wasting (PEW), a frequent form of malnutrition in patients with CKD, characterized by nutritional and metabolic alterations, is also prevalent in those on HD (7). In a Chinese cohort, 44.9% of patients on HD were diagnosed with PEW, which was independently associated with plasma fibroblast growth factor-23 (FGF-23) and Klotho, even after adjusting for covariables, highlighting their value as predictors of PEW in this population (Zhou et al.).

In summary, this Research Topic on Sarcopenia and Nutrition in CKD raises the complexity and clinical relevance of this condition by integrating evidence on key risk and protective factors, emerging tools for risk stratification and assessment, and the adverse outcomes associated with sarcopenia and malnutrition/PEW. These findings reinforce the need for early screening, accurate diagnosis, and more comprehensive approaches in this patient population.
